# Scintigraphic Investigations of the Deep and Superficial Lymphatic Systems in the Evaluation of Lower Limb Oedema

**DOI:** 10.1038/s41598-019-49554-7

**Published:** 2019-09-23

**Authors:** Romain Barbieux, Mirela Mariana Roman, Fanny Rivière, Olivier Leduc, Albert Leduc, Pierre Bourgeois, Steven Provyn

**Affiliations:** 1 Department of Nuclear Medicine and Multi-disciplinary Clinic-Unit of Lymphology, Institut Jules Bordet, Université Libre de Bruxelles, Brussels, Belgium; 2 Group R&D Clinical Applications of Fluorescence Imaging (GCAFI), Université Libre de Bruxelles, Brussels, Belgium; 3 Department of Mammo-Pelvic Surgery, Institut Jules Bordet, Université Libre de Bruxelles, Brussels, Belgium; 4 Lympho-phlebology Unit, Department of Occupational and Environmental Physiology, Haute Ecole HE2B ISEK, Brussels, Belgium; 5 Physiotherapy and rehabilitation department, Université Libre de Bruxelles, Brussels, Belgium; 6 Anatomical Research and Clinical studies, Vrije Universiteit Brussel, Brussels, Belgium

**Keywords:** Physical examination, Physical examination, Molecular imaging, Molecular imaging, Risk factors

## Abstract

The lymphoscintigraphic investigation (LySc) of the superficial lymphatic system (SLS) remains the gold standard for the diagnosis of lower limb lymphoedema. However, LySc of the deep lymphatic system (DLS) may be useful for diagnosing deep lymphatic system insufficiency in patients with lower limb oedema (LLE) but normal and/or paradoxical LySc of the SLS. The purpose of this study was therefore to evaluate a new LySc of the deep lymphatic system in patients presenting with a normal and/or paradoxical SLS exam showing LLE. In all, 15 patients with unilateral and 17 with bilateral LLE underwent 3-phased deep LySc of the lower limb via the injection of 99 mTc-labelled human serum albumin (HSA) nanocolloids in the Kager’s triangle. The absence of popliteal lymphatic node visualization after phase 2 of DLS LySc to diagnose a deep lymphatic insufficiency has a specificity and a sensitivity of 89% in patients with unilateral LLE and without associated venous symptoms. An insufficiency of the DLS was observed in 67% of cases with unilateral LLE and 59% of patients with bilateral LLE of venous and/or lymphatic origin. In conclusion, the lymphoscintigraphic visualization of the popliteal lymphatic nodes after the injection of 99 mTc-labelled HSA nanocolloids in the Kager’s triangle seems to be an effective way to diagnose DLS insufficiency in patients with LLE but normal findings in the SLS.

## Introduction

Lymphoedema is a chronic and evolving disease that can cause massive morbidity and takes many forms depending on its origin and localization. It affects 140 to 250 million people worldwide, and 99% of this population develops lymphoedema after cancer treatment or parasitic infection^1^. Although a patient’s clinical history and medical examination can be sufficient to diagnose lymphoedema in some situations, lymphoscintigraphy can be useful in many cases and is considered the gold standard to establish a diagnosis of lymphoedema^2–9^. In patients with lower limb lymphoedema, the clinical symptoms are mainly confined to the superficial compartment. Therefore, most lymphoscintigraphic (LySc) investigations are performed by injecting the radiotracer subcutaneously (or intradermally) into the first interdigital space as this provides valuable information concerning the impaired physiological and/or abnormal morphological characteristics of the superficial (or epifascial) lymphatic system (SLS). The human lymphatic system is subdivided into two different networks: the SLS and the deep (or subfascial) lymphatic system (DLS). In some patients with limb oedema, the investigation of the SLS can be normal, whereas other patients paradoxically present an SLS that functions better at the level of the oedematous limb than at the level of the contralateral healthy limb. In these cases, the patient’s clinical data and/or exam may lead to a diagnosis of a venous pathology (deep venous thrombosis, chronic venous insufficiency, etc.). Such venous problems can indeed lead to an overload of the superficial lymphatic system^3,10–13^. However, those venous problems may also be absent, and a clinical history may suggest a lymphoedema of primary origin (for instance, typically in young women with unilateral symptoms and a family history of lower limb oedema)^14^. Based on the results of radiological lymphangiographies, Tosatti reported in 1974 that some cases of lymphoedema (those affecting only the dorsum of the foot and the ankle) were related to an impairment of the DLS but not the SLS^15^. In 1993, Bräutigam *et al*. proposed a protocol for two-compartment lymphoscintigraphy^11^ and confirmed in 1998 that an investigation of the DLS might be necessary to understand the physiopathology of certain types of complex oedema^14^. The purpose of this study was to evaluate a methodological approach of evaluating lower limb DLS in patients presenting with unilateral or bilateral lower limb oedema (LLE) with no sign of morphological or functional abnormalities in the SLS.

## Materials and Methods

This is a retrospective study based on the analysis of DLS LySc in patients treated at the Jules Bordet Institute, Brussels, Belgium. All medical data and information regarding the patients included in this study were used in agreement with the rules of conduct dictated by the institution and in agreement with the ethics committee of the Jules Bordet Institute (ethics committee number 2048).

Every LySc was performed by the same investigator following a lower limb superficial LySc protocol^16^ approved by the Belgian Society of Nuclear Medicine^17^ and recognized by the Belgian National Health Insurance System (Institut National Assurance Maladie Invalidité or INAMI). The DLS protocol corresponded to an adapted version of the SLS protocol itself, which was established by the SLS protocol founder. This adaptation was necessary to take into account the anatomical and physiological differences between the DLS and the SLS.

To be included in this study, all patients met the following criteria:

1. presented with either unilateral LLE or bilateral asymmetric LLE,
2. had prior superficial LySc of the lower limbs with neither functional abnormalities (normal tracer progression at rest and with muscular contractions) nor morphological abnormalities (dermal backflow presence, absence of inguinal and/or iliac lymphatic nodes) in the SLS., and
3. (at least two days later) deep LySc of the lower limbs.

Patients with primary lower limb lymphoedema and abnormal function of the superficial lymphatic system were excluded from the present study because Bräutigam *et al*. reported in 1993 that these patients also exhibit abnormal function of their deep lymphatic system^11^.

The lymphoscintigraphical imaging data of 27 female and 5 male patients (aged from 14 to 74 years old) obtained between March 2010 and December 2015 were analysed for this study (Table 1).

**Table 1:** Population analyses table.

	Group 1	Group 2
N	15	16
Women	11	15
Men	4	1
Age (years)	40.5 ± 12.8	23.6 ± 14.5
Venous Oedema Origin	6	4
• Saphenectomy	• 2	• 3
• Deep venous thrombosis/Phlebitis	• 4	• 1
Lymphatic Oedema Origin	9	12
• Primary lymphoedema	• 6	• 10
• Secondary lymphoedema	• 3	• 0
• Lymphangitis	• 0	• 1
• Mycosis	• 0	• 1

The analysis was conducted in two separate groups:

Group 1 consisted of 15 patients with unilateral LLE with a lymphatic and/or venous origin. Group 2 consisted of 17 patients with asymmetric bilateral LLE from a lymphatic and/or venous origin.

Before every LySc, patients received instructions regarding the protocol and the importance of following it. Prior to the exam, patients were asked to remove any elastic stockings. Patients were then positioned in dorsal decubitus on the examination bed, and the injections were administered after at least 5 minutes of rest. Afterwards, lymphoscintigraphy was conducted with an SMV ST-XLi from GE Healthcare (GE-Healthcare, Little Chalfont, United Kingdom).

The solution injected for both SLS and DLS LySc was made by adding 30 mCi (1110 MBq) of oxido(trioxo)technetium (TcO_4_^−^) to a vial of Human Serum Albumin nanosized colloids Nanocoll® (GE Healthcare, Little Chalfont, United Kingdom); 0.9% saline was then added to reach a final volume of 2.0 ml. A volume of 0.2 cc (3 mCies per syringe) of this final solution was injected into each foot.

The SLS LySc injections were administered subcutaneously in the first interdigital space of each lower limb. The DLS LySc injections were administered in the retro malleolar anterior space of the triceps surae tendon (Kager’s triangle) to select the deep lymphatic drainage, and the tracer was injected subfascially. Both LySc protocols were subdivided into 4 phases as described in Table 2 for the SLS LySc and in Table 3 for the DLS LySc. Every LySc was analysed by the same expert investigator and verified by a second investigator. Each phase of the protocol was set to evaluate one physiological aspect of the patient’s lymphatic drainage, and an additional phase 0 provided the possibility to further quantify the tracer extraction following each phase, as follows:

**Table 2:** Imaging protocol for lymphoscintigraphical investigation of the lower limb superficial lymphatic system.

Phase n°	Description	Acquisition parameters
***Superficial lymphatic system lymphoscintigraphical investigation***		
0	Camera centred on the injection site and the syringe used for radioactivity measurement	Anterior static imaging: Word mode, Matrix 128 × 128, 60 sec
1	Patient at rest for 30 min Dynamic imaging centred on the inguinal area Whole-body scanning performed after dynamic imaging	Anterior dynamic imaging: Byte mode, Matrix 64 × 64, 90 frames over 20 sec
		Antero-posterior whole-body scanning: Step and shoot mode, 6 steps, 2048 × 512, 34 cm/min
2	Lower limbs at rest during 5 min followed by 5 min of active plantar and dorsal alternate flexion of the ankle followed by 5 min of rest Dynamic imaging centred on the inguinal area Whole-body scanning performed after dynamic imaging	Anterior dynamic imaging: Byte mode, Matrix 64 × 64, 90 frames over 10 sec
		Antero-posterior whole-body scanning: Step and shoot mode, 6 steps, 2048 × 512, 34 cm/min
3	The patient was required to walk for a full hour Static imaging camera centred on the injection site after the walk Whole-body scanning performed after static imaging	Anterior static imaging: Word mode, Matrix 128 × 128, 60 sec
		Antero-posterior whole body scanning: Step and shoot mode, 6 steps, 2048 × 512, 34 cm/min

General Parameters: Collimator, low-energy with ultra-high resolution 140 keV Energy window of 20% Zoom 1.

**Table 3:** Imaging protocol for lymphoscintigraphical investigation of the lower limb deep lymphatic system.

Phase n°	Description	Acquisition parameters
***Deep lymphatic system lymphoscintigraphical investigation***		
0	Camera centred on the injection site and the syringe used for radioactivity measurement	Antero-posterior static imaging: Word mode, Matrix 128 × 128, 60 sec
1	Patient at rest for 30 min Dynamic imaging centred on the inguinal area during the phase First static imaging centred on the calves after dynamic imaging Second static imaging centred on the inguinal area after the previous static imaging	Antero-posterior dynamic imaging: Byte mode, Matrix 64 × 64, 90 frames over 20 sec
		Antero-posterior static imaging: Word mode, Matrix 128 × 128, 60 sec
2	Patient performed active plantar and dorsal flexion of the ankle for a period of 15 min Dynamic imaging centred on the popliteal area during the phase Static imaging first centred on the calves and then on the inguinal area	Antero-posterior dynamic imaging: Byte mode, Matrix 64 × 64, 90 frames of 10 sec
		Antero-posterior static imaging: Word mode, Matrix 128 × 128, 60 sec
3	The patient was required to walk for a full hour Static imaging camera centred on the injection site after the walk Static imaging was first centred on the calves and then on the inguinal area	Antero-posterior static imaging centred on the injection site: Word mode, Matrix 128 × 128, 60 sec
		Antero-posterior static imaging centred on the calves and inguinal area: Word mode, Matrix 128 × 128, 120 sec

General Parameters: Collimator with low-energy ultra-high resolution 140 keV Energy window of 20% Zoom 1

Phase 1: Evaluation of the lymphatic drainage after a period of rest,

Phase 2: Evaluation of the lymphatic drainage after a period of moderate physical activity (5 minutes of tiptoeing), and

Phase 3: Evaluation of the lymphatic drainage after a longer period (one hour) of activity similar to that used in daily activities.

Statistical analysis were performed with GraphPad using contingency tables with Fisher’s exact tests and Chi-square tests to verify the possible dependencies between:

1. The presence of LLE and visualization of the popliteal lymphatic nodes (PLNs) during each phase of DLS LySc in the population with unilateral LLE with non-pathological SLS (group 1),
2. The cause of the LLE and the visualization of the PLNs on DLS LySc in the population with unilateral LLE with non-pathological SLS (group 1), and
3. PLN visualization of both lower limbs on DLS LySc in the population with asymmetric bilateral LLE with non-pathological SLS (group 2).

Statistical significance was determined as follows:

A p value superior or equal to 0.05 indicated no significance was reached, and the null hypothesis was not rejected.

A p value between 0.05 and 0.01 or equal to 0.01 indicated that significance was reached, and the null hypothesis was rejected.

A p value between 0.01 and 0.001 or equal to 0.001 indicated that high significance was reached, and the null hypothesis was rejected.

A p value lower than 0.001 indicated that the highest significance was reached, and the null hypothesis was rejected.

## Results

### Dependency between the presence of LLE and the visualization of PLNs during each phase of DLS LySc in the population with unilateral LLE and non-pathological SLS

**Figure 1:**
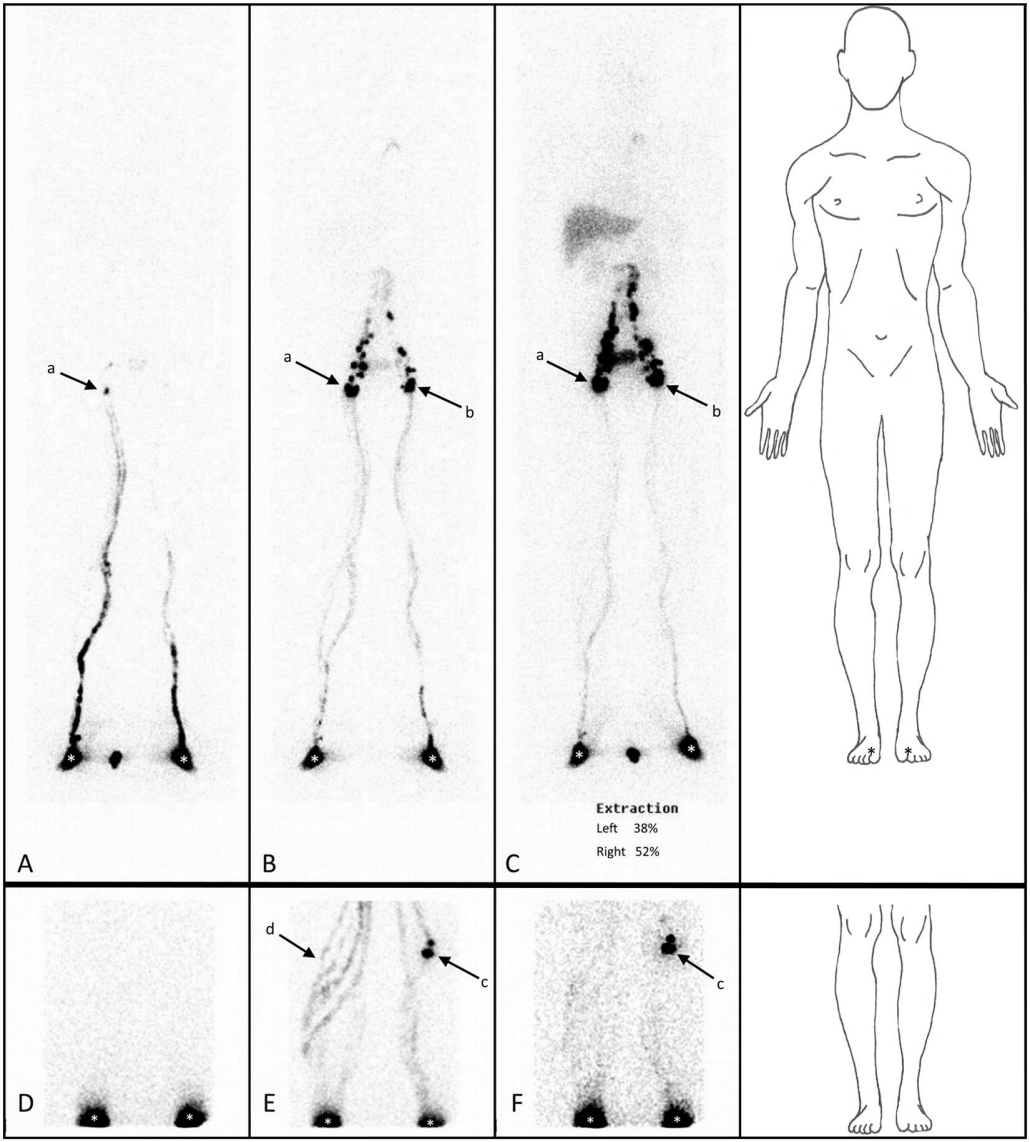
Anterior LySc of SLS drainage after phase 1 (**A**), phase 2 (**B**) and phase 3 (**C**) and DLS drainage after phase 1 (**D**), phase 2 (**E**) and phase 3 (**F**) in a patient with oedema of the right lower limb (injection sites are indicated by a white *). LySc of the SLS showing neither functional nor morphological signs of lower limb lymphoedema and the appearance of right inguinal lymphatic nodes (a) following the first phase and left inguinal lymphatic nodes (b) following the second phase. In contrast, the LySc of the lower limbs DLS allowed the visualization of the left PLNs (c) following the second phase, whereas the right lower limbs showed a diversion of the lymphatic flow from the DLS to the SLS without visualization of the right PLNs as a sign of DLS insufficiency.

The first phase showed no dependency between the imaging of PLNs and the presence of oedema. However, PLNs were observed in 93% (14/15) of healthy lower limbs versus 33% (5/15) of oedematous lower limbs after the 2^nd^ phase and in 93% (14/15) of healthy lower limbs versus 47% (7/15) of oedematous lower limbs after the 3^rd^ phase. This dependency was statistically significant for phase 2 and phase 3 (See Table 4). An example of LySc for the population with unilateral LLE and with non-pathological SLS is shown in Fig. 1.

**Table 4:** Contingency table representing the dependency between the PLN visualization and the presence of oedema for the population of patients presenting a unilateral lower limb oedema after deep lymphatic system lymphoscintigraphy.

		PLN+	PLN−	Total	p value χ²	p value Fisher
***Unilateral lower limb oedema***						
Phase 1	HLL	4	11	15	0.142	0.330
	ELL	1	14	15		
	Total	5	25	30		
**Phase 2**	**HLL**	**14**	**1**	**15**	**<0.001**	**0.002**
	**ELL**	**5**	**10**	**15**		
	**Total**	**19**	**11**	**30**		
Phase 3	HLL	14	1	15	0.005	0.014
	ELL	7	8	15		
	Total	21	9	30		

PLN+: Popliteal lymphatic node visualization; PLN−: No popliteal lymphatic node visualization; ELL: Oedematous lower limb; HLL: Healthy lower limb.

As shown in Table 4, the best combination for establishing insufficiency of the DLS in terms of specificity, sensitivity, positive predictive value and negative predictive value was obtained after phase 2. Figure 1 shows examples of superficial and deep LySc in one of the patients who presented with unilateral lower limb oedema (See Table 5) (insert Fig. 1).

**Table 5:** Analysis of characteristics of the PLN criteria for DLS lymphoscintigraphical assessment in the population with unilateral lower limb oedema.

	Sensitivity	Specificity	+PV	−PV
***Unilateral lower limb oedema***				
Phase 1	93%	27%	56%	80%
**Phase 2**	**67%**	**93%**	**91%**	**74%**
Phase 3	53%	93%	89%	67%

PLN: Popliteal lymphatic node; +PV: Positive predictive value; −PV: Negative predictive value.

### Dependency between the cause of the LLE and the visualization of PLNs on DLS LySc in the population with unilateral LLE with non-pathological SLS

In our population of 15 subjects with unilateral LLE but non-pathological SLS, PLNs were statistically more frequently visualized at the level of the LLE with a venous origin than at the level of LLE with a lymphatic origin after phase 2 but not after phase 3 (see Table 6). However, statistical significance was only reached with the χ² test, mostly because of the small number of cases.

**Table 6:** Contingency table representing the dependency between PLN visualization and the cause of oedema in the population of patients presenting unilateral lower limb oedema after deep lymphatic system lymphoscintigraphy.

		PLN+	PLN−	Total	p value χ²	p value Fisher
***Unilateral lower limb oedema***						
**Phase 2**	**Venous**	**4**	**2**	**6**	**0.025**	**0.089**
	**Lymphatic**	**1**	**8**	**9**		
	**Total**	**5**	**10**	**15**		
Phase 3	Venous	4	2	6	0.205	0.315
	Lymphatic	3	6	9		
	Total	7	8	15		

PLN+: Popliteal lymphatic node visualization; PLN−: No popliteal lymphatic node visualization.

Among the population with unilateral LLE without associated venous symptoms (n = 9), PLNs were seen in 89% of the healthy lower limbs versus 11% of the oedematous lower limbs after the 2^nd^ phase and in 89% of the healthy lower limbs versus 33% of the oedematous lower limbs after the 3^rd^ phase. Statistical significance was reached following the second and third phases (see Table 7).

**Table 7:** Contingency table representing the dependency between PLN visualization and the presence of oedema in the population of patients presenting with unilateral lower limb oedema without associated venous symptoms after deep lymphatic system lymphoscintigraphy.

		PLN+	PLN−	Total	p value χ²	p value Fisher
***Unilateral lower limb oedema with a lymphatic cause***						
**Phase 2**	**HLL**	**8**	**1**	**9**	**0.001**	**0.0034**
	**ELL**	**1**	**8**	**9**		
	**Total**	**9**	**9**	**18**		
Phase 3	HLL	8	1	9	0.016	0.0498
	ELL	3	6	9		
	Total	11	7	18		

PLN+: Popliteal lymphatic node visualization; PLN−: No popliteal lymphatic node visualization; ELL: Oedematous lower limb; HLL: Healthy lower limb.

The results obtained in the population with unilateral LLE without associated venous symptoms confirmed what was observed in the general population with unilateral LLE. As shown in Table 8, the best combination for establishing insufficiency of the DLS in terms of specificity, sensitivity, positive predictive value and negative predictive value was obtained after phase 2 (see Table 8).

**Table 8:** Analysis of PLN characteristics as criteria for DLS lymphoscintigraphical assessment in the population with unilateral lower limb oedema without associated venous symptoms.

	Sensitivity	Specificity	+PV	−PV
***Unilateral lower limb oedema with a lymphatic cause***				
**Phase 2**	**89%**	**89%**	**89%**	**89%**
Phase 3	67%	89%	86%	73%

PLN: Popliteal lymphatic node; +PV: Positive predictive value; −PV: Negative predictive value.

### Dependency between PLN visualization in both lower limbs and DLS LySc in the population with asymmetric bilateral LLE with non-pathological SLS

**Figure 2:**
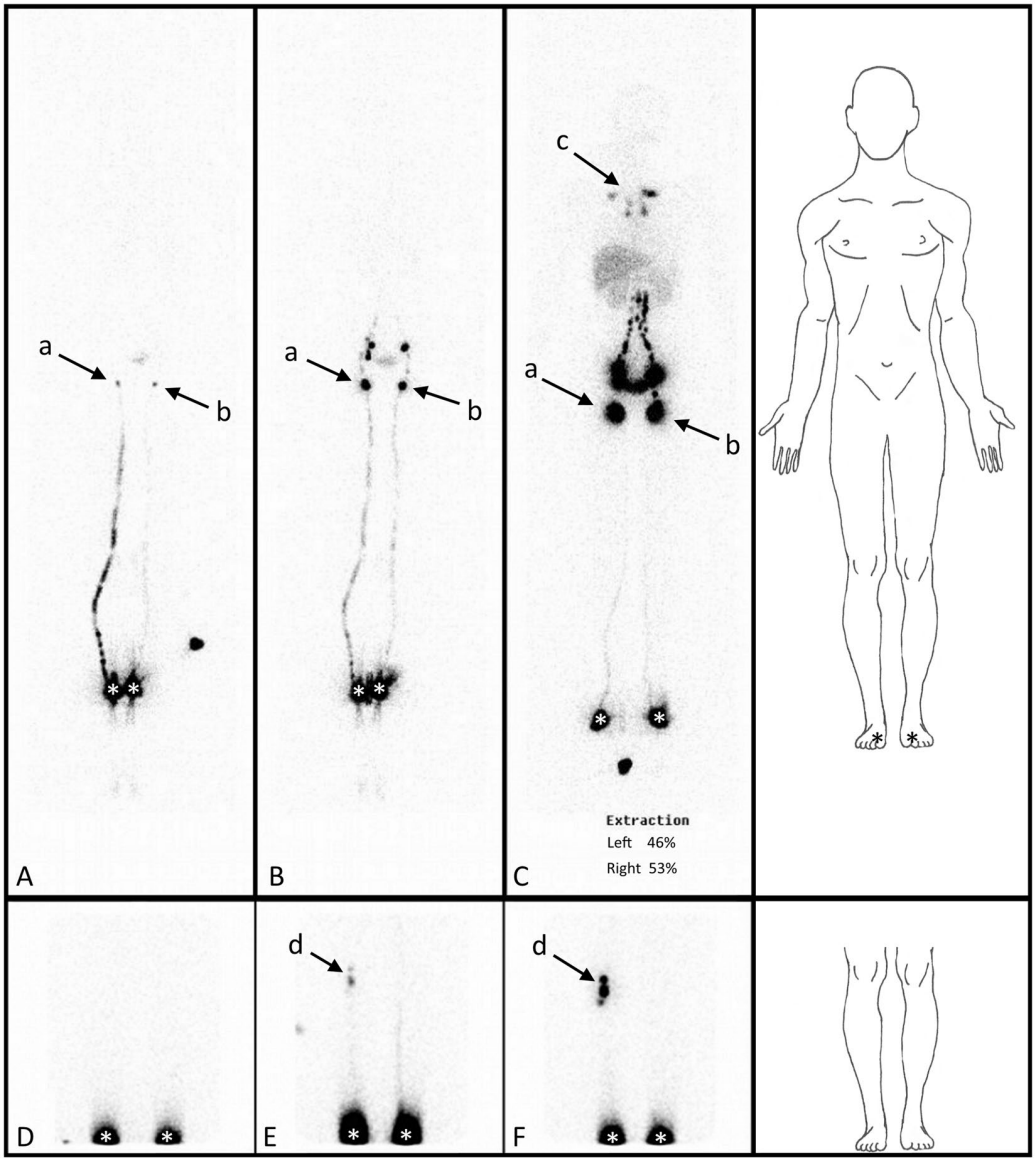
Anterior LySc of SLS drainage after phase 1 (**A**) phase 2 (**B**) and phase 3 (C) and DLS drainage after phase 1 (**D**), phase 2 (**E**) and phase 3 (**F**) in a patient with left dominant asymmetric bilateral oedema of the lower limbs (injection sites are indicated by a white *). LySc of the SLS showed neither functional nor morphological signs of lower limb lymphoedema with the appearance of right (a) and left (b) inguinal lymphatic nodes following the first phase even in the absence of a malformation of the thoracic duct. (c) These findings were suggestive of primary lymphoedema. In contrast, while the LySc of the lower limbs DLS allowed the visualization of the right PLNs (d) following the second phase, the right lower limbs showed no signs of PLN visualization and were considered a sign of DLS insufficiency.

In the 17 patients with bilateral LLE, PLNs were observed more frequently (see Table 9) after the second and third phases of DLS LySc at the level of the less swollen limb than at the level of the more swollen limb. The dependence between the importance of the oedema and the presence or absence of PLNs on DLS LySc shows that this approach is more likely to find deep lymphatic insufficiency when extensive oedema is present. This confirms our previous results showing that at least a portion of LLE may be due to deep lymphatic insufficiency and that the importance of that oedema could depend on the extent of DLS impairment. An example of the results of LySc obtained among the population with asymmetric bilateral LLE with non-pathological SLS is shown in Fig. 2.

**Table 9:** Contingency table representing the dependency between PLN visualization and the presence of oedema in the population of patients presenting with asymmetric bilateral lower limb oedema after deep lymphatic system lymphoscintigraphy.

		PLN+	PLN−	Total	p value χ²	p value Fisher
***Bilateral asymmetric lower limb oedema***						
Phase 1	OM−	2	15	17	0.1449	0.4848
	OM+	0	17	17		
	Total	2	32	34		
Phase 2	OM−	14	3	17	0.0313	0.0707
	OM+	8	9	17		
	Total	22	12	34		
Phase 3	OM−	15	2	17	0.0239	0.0570
	OM+	9	8	17		
	Total	24	10	34		

PLN+: Popliteal lymphatic node visualization; PLN−: No popliteal lymphatic node visualization; OM+: Most oedematous lower limb; OM−: Less oedematous lower limb.

Finally, the results showed that among the 17 patients in whom no lymphoedema was diagnosed based on SLS LySc, 10 (59%) had lower limb DLS insufficiency, including 2 cases in which it was bilateral and 8 in which it was unilateral (Insert Fig. 2).

## Discussion

Most of the time, and particularly when using near-infrared fluorescence imaging techniques that allow only the visualization of the SLS^18^, imaging investigations of LLEs suspected to be of lymphatic origin are solely based on lymphoscintigraphic examinations (LySc) of the SLS^19^. However, the importance of the DLS in cases of LLE has been questioned in past literature studies that included small series of patients with various sites of tracer injection^11,12,14,20,21^.

In previous DLS scintigraphic investigations, intramuscular injection in the distal third of the calf^12^, deep injection in the sole of the foot^11,14^ or superficial injection in the external retro-malleolar space^20^ have both been proposed. However, whereas the two first injections (intra-muscular and in the sole of the foot) allow visualization of the deep lymphatic system of the calf, the latter does not. These studies also evaluate DLS drainage through the quantification of activities in the inguinal LNs, which seems somewhat illogical. Part of the tracer is indeed trapped in the popliteal LNs before it reaches the inguinal LNs, and the activities observed in the inguinal LNs are not completely representative of the tracer removed by the lymphatic system at the level of the injected site.

In our study, the use of LySc of the DLS to visualize PLNs following subfascial injection in Kager’s triangle (limited posteriorly by the triceps surae tendon, anteriorly by the posterior side of the tibia and inferiorly by the superior side of the calcaneus) is proposed for the first time. With this subfascial injection, the tracer is drained specifically by the DLS, and considering our results, the visualization of PLNs after injection of the tracer in Karger’s triangle seems to be effective in the diagnosis of DLS insufficiency among oedematous patients with a normal LySc in the SLS.

In this study, our analysis of 15 patients with unilateral LLE showed that only for one of these patients, no PLNs was imaged at the level of the healthy lower limb after the second phase of DLS LySc. This suggests that after the second phase, the visualization of the PLNs on DLS imaging represents a specific criterium for the normality of the DLS. With a positive and negative predictive value of almost 90% in the population of patients with lymphatic causes, we consider this criterium relevant, even if the series assessed was rather small. In doing so, we found that 67% (10/15) of the patients with unilateral LLE and 58% (10/17) of the patients with bilateral LLE showed no popliteal LNs on DLS imaging of their oedematous lower limb, suggesting that DLS insufficiency and a lymphoedematous situation were present in 63% (20/32) of the patients in the whole series (of the patients who presented with symptoms of oedema but had a normal LySc of the SLS). Because every patient included in this study underwent previous SLS LySc, those 20 cases can be considered false-negatives for SLS LySc that became true positives for DLS LySc. This means that if the patient examination had been stopped after the SLS LySc, these 20 patients would have left the hospital without any diagnosis, no treatment would have been proposed, and they would have had an impaired quality of life due to their false-negative diagnosis.

More specifically, if we limit the analysis of the patients with unilateral LLE to cases without any prior venous problems, this investigation of the deep lymphatic system was abnormal in 8 out of 9 cases, suggesting a sensitivity of 89% for this approach to detect oedema related to primary insufficiency of the deep lymphatic system. These data can only be partially compared to the data published by Bräutigam *et al*. in 1993^11^. Those authors reported on the superficial and deep compartment in female patients with primary lymphoedema. Five of their patients showed normal SLS function (our inclusion criteria), and 3 out of these five had reduced DLS function. However, of these 3 patients, 2 had persistent lymphoedema and 1 had intermittent oedema; in the 2 patients with normal SLS and normal DLS, there were no clinical signs of lymphoedema at the time of their examination.

In contrast, if we limit the analysis of patients with unilateral LLE to cases with prior venous problems, our investigation of the deep lymphatic system would have been normal in 4 out of 6, or 66% of the cases, consistent with the data in the literature. The situation in patients with antecedents of venous problems is more difficult to analyse because venous oedema can lead to lymphatic insufficiency^22^. Patients with LLE of venous origin could then present with functional (and/or morphological) impairment not only due to their SLS but also due to the DLS (due to impaired functions that may be masked by an overload related to venous problems). In 1978, Tosatti used radiological lymphangiography and found that in 77% of patients with post-phlebitic syndrome, the DLS showed an increase in the number and calibre of lymphatic vessels without signs of lymphatic insufficiency^23^. In 1994, Partsch and Mostbeck reported that subfascial lymph transport was decreased not only in the post-thrombotic stage but also in the acute phase of deep vein thrombosis^24^. They observed an absence of LN visualization (inguinal and/or popliteal) in 41% of the cases in addition to frequently decreased abnormal DLS function combined with an increasing delay after the acute thrombotic event. Bräutigam *et al*. (1998) reported that the function of the DLS was normal in all cases with venous oedema (7/7) but abnormal in all (11/11) patients with post-thrombotic syndrome (6 had an abnormal evaluation in the SLS)^14^.

This study is retrospective, and the number of cases may be considered rather small. However, the subjects corresponded to a particular type of patient, and the site of injection has not previously been reported in the literature. Despite these limitations, we think that these results might have implications in clinical practice. In cases of lymphoedema, an early diagnosis is important to prevent complications, such as infections, loss of mobility, tiredness and psychological distress^25^. The diagnosis of an insufficient DLS might consequently modify the management of these patients. The classical physical treatment strategy is based on increasing the SLS lymphatic flow^26^ and the liquid resorption. In contrast, in our population, the SLS showed no sign of lymphatic insufficiency, and physical treatment should therefore be adapted to treat the DLS insufficiency. As shown in our results, normal deep lymphatic drainage should be observed on the DLS LySc after 15 minutes of moderate physical activity. However, if the second phase is sufficient to diagnose DLS insufficiency following a phase of more intensive physical activity, we can observe PLNs in 4 of the 32 oedematous lower limbs (after phase 3). If this represents only 12.5% of our series, this is showing that physical activity could help these patients by having affecting the physical treatment for DLS insufficiency by increasing or forcing the resorption. Indeed, most cases of lymphedema are due to an SLS insufficiency which justifies that the physical treatment prescribed in such cases are based on superficial technics with low pressure like manual lymphatic drainage in order to stimulate the peripheral resorption and increase the lymphatic flow in the SLS. However, most of the cases investigated in this study are showing no SLS insufficiency, and this could suggest that the classical physical treatment could be inefficient. Therefore, it seems important for both the patient and the physical therapist to know which type of insufficiency is causing the symptoms so that the treatment can be oriented to increase its efficiency. Specific physical exercises or deeper massage techniques could be more efficient in stimulating DLS in such patients. We advise that the possible ways that lymphoedema could be treated due to DLS insufficiency should be investigated in future clinical trials. Indeed, in 2016, a study by Robert Weiss showed that proper physical treatment for lymphoedema resulted in lower medical costs and fewer hospitalizations^27^. This showed that obtaining a diagnosis of DLS insufficiency through DLS LySc could lead to a reduction in healthcare costs while increasing patient quality of life because it allows more specific and personalized treatment.

In conclusion, the results suggest that for some patients, an LLE could be caused by an impairment of their DLS, making the DLS LySc the best way to diagnose the true cause of their LLE. This is important so that the patient can know the origin of his or her symptoms, and it has also been demonstrated that an early diagnosis leads to more efficient physical treatment^28^. From a practical point of view, DLS LySc of the SLS should be indicated in patients with unilateral or bilateral LLE (or the sensation of LLE) but without morphological or functional signs of lymphatic insufficiency whose history and clinical examination are suggestive of the presence of lymphoedema.
